# Protocol for the PACE trial: A randomised controlled trial of adaptive pacing, cognitive behaviour therapy, and graded exercise as supplements to standardised specialist medical care versus standardised specialist medical care alone for patients with the chronic fatigue syndrome/myalgic encephalomyelitis or encephalopathy

**DOI:** 10.1186/1471-2377-7-6

**Published:** 2007-03-08

**Authors:** Peter D White, Michael C Sharpe, Trudie Chalder, Julia C DeCesare, Rebecca Walwyn, the PACE trial group

**Affiliations:** 1 Department of Psychological Medicine, Queen Mary School of Medicine and Dentistry, St Bartholomew's Hospital, London, UK; 2 Psychological Medicine and Symptoms Research Group, University of Edinburgh, Royal Edinburgh Hospital, Edinburgh, Scotland, UK; 3 Academic Department of Psychological Medicine, Guy's, King's and St Thomas' School of Medicine, Weston Education Centre, London, UK; 4 PACE Trial Coordinating Centre, Queen Mary School of Medicine and Dentistry, St Bartholomew's Hospital, London, UK; 5 Mental Health & Neuroscience Clinical Trials Unit (MH&N CTU), Institute of Psychiatry, London, UK

## Abstract

**Background:**

Chronic fatigue syndrome (CFS, also called myalgic encephalomyelitis/encephalopathy or ME) is a debilitating condition with no known cause or cure. Improvement may occur with medical care and additional therapies of pacing, cognitive behavioural therapy and graded exercise therapy. The latter two therapies have been found to be efficacious in small trials, but patient organisations' surveys have reported adverse effects. Although pacing has been advocated by patient organisations, it lacks empirical support. Specialist medical care is commonly provided but its efficacy when given alone is not established. This trial compares the efficacy of the additional therapies when added to specialist medical care against specialist medical care alone.

**Methods/Design:**

600 patients, who meet operationalised diagnostic criteria for CFS, will be recruited from secondary care into a randomised trial of four treatments, stratified by current comorbid depressive episode and different CFS/ME criteria. The four treatments are standardised specialist medical care either given alone, or with adaptive pacing therapy or cognitive behaviour therapy or graded exercise therapy. Supplementary therapies will involve fourteen sessions over 23 weeks and a 'booster session' at 36 weeks. Outcome will be assessed at 12, 24, and 52 weeks after randomisation. Two primary outcomes of self-rated fatigue and physical function will assess differential effects of each treatment on these measures. Secondary outcomes include adverse events and reactions, subjective measures of symptoms, mood, sleep and function and objective measures of physical activity, fitness, cost-effectiveness and cost-utility. The primary analysis will be based on intention to treat and will use logistic regression models to compare treatments. Secondary outcomes will be analysed by repeated measures analysis of variance with a linear mixed model. All analyses will allow for stratification factors. Mediators and moderators will be explored using multiple linear and logistic regression techniques with interactive terms, with the sample split into two to allow validation of the initial models. Economic analyses will incorporate sensitivity measures.

**Discussion:**

The results of the trial will provide information about the benefits and adverse effects of these treatments, their cost-effectiveness and cost-utility, the process of clinical improvement and the predictors of efficacy.

## Background

### Introduction

The chronic fatigue syndrome (CFS) is a condition characterised by chronic disabling fatigue and other symptoms, which are not better explained by an alternative diagnosis [1-3]. Myalgic encephalomyelitis/encephalopathy (ME) refers to a severe debilitating illness thought by some to be a separate illness, but by others to be synonymous with CFS [2-6]. In keeping with the MRC Research Advisory Group report and the CMO's working group report, we will refer to the illness using both terms: CFS/ME [4,6]. The prevalence of CFS/ME in the population is between 0.4 and 2.5% [3,4,6]. A working group, reporting to the Chief Medical Officer (CMO) for England, concluded; "CFS/ME is a relatively common clinical condition, which can cause profound, often prolonged, illness and disability, and can have a substantial impact on the individual and the family" [4]. As many as half the patients with CFS/ME are unemployed [7], and they have 10 times the amount of sick-leave of other general medical outpatients [8]. The prognosis is poor: in primary care only a third improve by one year, and of those referred to secondary care less than 10% return to pre-morbid functioning [3,9]. The management of patients with CFS/ME currently consumes significant resources in both primary and secondary care with uncertain benefit to patients [4,5]. CFS/ME patients use an annual average of 13 visits to their general practitioner and 5 visits to secondary care [7]. There is now some evidence that specific treatments can improve these poor outcomes. The CMO's working group concluded; "Therapeutic strategies that can enable improvement include graded exercise/activity programmes, cognitive behaviour therapy, and pacing" [4]. However this positive statement was balanced in the report by other statements: first the concern of patient organisations that graded exercise therapy (GET) may sometimes worsen symptoms and disability, and second that pacing, although widely advocated by patients' organisations, is as yet unsupported by scientific evidence.

### Efficacy – Relevant studies/trials

Two independent systematic reviews have found that rehabilitative cognitive behaviour therapy (CBT) and GET were the most promising treatments for CFS/ME in secondary care [5,10-12]. The published trials of these treatments were however also criticized for being too small, too selective, and for using different outcome measures. No other treatments for CFS/ME have so far been shown to be helpful in more than one RCT [5,12]. CBT is a more complex therapy than GET, requiring highly trained therapists, and is therefore less readily available. In contrast, surveys carried out by *Action for M.E. *of their members have indicated that CBT and GET can sometimes make people worse [13-15]. Pacing and rest were reported to be more helpful [13]. Pacing has been described in the scientific literature as a lifestyle management that allows optimal adaptation to the illness, including an appropriate balance of rest and activity [4,16]. It has been advocated by exponents of the "envelope theory" of CFS/ME, which states that a patient has a fixed and finite amount, or "envelope", of energy that they must adapt to by managing their activity [16]. A non-randomised comparison of adaptive (rather than rehabilitative) CBT, which included adaptive pacing therapy (APT) based on this model, found that, although fatigue improved, this treatment was no more effective than the control treatment in reducing disability [17]. A recent systematic review concluded that there was insufficient evidence to recommend APT at present [5,10,12]. In a similar way there is little RCT evidence of the efficacy of specialist medical care. There is therefore an urgent need to: (a) compare the supplementary therapies of both CBT and GET with both APT and standardised specialist medical care (SSMC) alone, seeking evidence of both benefit and harm (b) compare supplementary APT against SSMC alone and (c) compare the supplementary therapies of APT, CBT and GET in order to clarify differential predictors and mechanisms of change.

### Differential outcomes

Because CBT and GET are both based on a graded exposure to activity, they may preferentially reduce disability, whilst APT, being based on the theory that one must stay within the limits of a finite amount of "energy", may reduce symptoms, but at the expense of not reducing disability. By measuring both symptoms and disability as our primary outcomes, we will be able to test a secondary hypothesis that these treatments may differentially affect symptoms and disability.

### Process of treatment

We do not know the mechanisms of successful treatment for CFS/ME. Do illness beliefs or focusing of attention on symptoms (symptom focusing) need to be changed for CBT to be effective? Or do CBT and GET both work by improving tolerance to activity? Is increased physical fitness essential to recovery or not? How important is the alliance between therapist and patient? Is it necessary to adapt to the limitations imposed by the illness to reduce fatigue? A greater understanding of these processes will shed light on the essence of improvement and allow the development of more efficient treatments.

### Predictors of outcome

Predictors of a negative response to treatment found in previous studies include having a mood disorder, membership of a self-help group, being in receipt of a disability pension, focusing on physical symptoms, and pervasive inactivity [3,18,19]. There is however no general agreement on which are the most important predictive factors.

### Cost-effectiveness and cost utility

A recent study has suggested that there is little difference in the cost-effectiveness of CBT and GET for chronic fatigue in primary care, and both were more expensive *and *more effective than standard care [20]. However, only one-third of patients in this study had CFS/ME and it was not powered to detect differences for this subgroup. There are currently only limited published data on the cost-effectiveness of treatments specifically for CFS/ME.

### Risks and benefits

There is a discrepancy between surveys of CFS/ME patient group members and published evidence from trials. Some CFS/ME charity members have reported that they feel worse after exercise therapy, and to a lesser extent CBT [13,14], whereas the trial evidence suggests minimal or no risk with these treatments. A further survey by *Action for M.E. *of their members suggests that reports of deterioration with therapy are related to either poorly administered treatment or lack of appropriate professional supervision [15]. The individual treatment programmes used in PACE will minimise this risk by being mutually agreed between participant and therapist, carefully monitored and flexibly implemented. We will also carefully monitor all participants for any adverse effects of the treatments, and will undertake a detailed assessment, at home if necessary, of any participant who reports deterioration or who withdraws from treatment, following which they will be offered appropriate help.

### Rationale

The results of this trial will: (a) allow people with CFS/ME, clinicians and health planners to choose treatment on the basis of both efficacy and cost; (b) provide evidence about the efficacy and adverse effects of the four treatments (APT, CBT, GET and SSMC); (c) provide the first test of SSMC plus pacing against SSMC alone; (d) indicate which patient characteristics predict a successful outcome; (e) identify which patient characteristics predict response to which treatment and (f) define the essential aspects of effective treatment as a first step toward the development of more efficient therapies.

The trial will recruit new patients from secondary care clinics run by three different disciplines (immunology, infectious disease and psychiatry) in six different centres in both England and Scotland. This recruitment plan will ensure sufficient heterogeneity to allow generalisation of the findings. We will not recruit directly from primary care because we wish to compare the efficacy of these treatments in patients whom GPs regard as requiring additional help and who are likely to have a worse prognosis (one of the recommendations CMO's report [4]). Furthermore, direct recruitment from primary care has been found to be problematic in previous studies. Two recent trials of treatment for prolonged fatigue (not CFS/ME) using large and well established primary care research networks recruited only 46 patients with CFS/ME in three years [21] and 44 patients in 2.5 years [22].

## Methods/Design

### Aims

The main aim of this trial is to provide high quality evidence to inform choices made by patients, patient organisations, health services and health professionals about the relative benefits, cost-effectiveness, and cost-utility, as well as adverse effects, of the most widely advocated treatments for CFS/ME.

The secondary aims of this trial are to investigate the mechanisms and predictors of a successful outcome.

### Objectives

The PACE trial is designed to answer the following questions:

#### Primary objectives

(1) Is APT and SSMC more effective than SSMC alone in reducing (i) fatigue, (ii) disability, or (iii) both?

(2) Is CBT and SSMC more effective than APT and SSMC in reducing (i) fatigue, (ii) disability or (iii) both?

(3) Is GET and SSMC more effective than APT and SSMC in reducing (i) fatigue, (ii) disability, or (iii) both?

(4) Are the *active *rehabilitation therapies (of either CBT or GET) more effective than the *adaptive *approach of APT when each is added to SSMC, in reducing (i) fatigue and/or (ii) disability?

(5) What are the relative cost-effectiveness and cost-utility of these treatments?

*NB For the sake of brevity, the rest of the protocol will refer to the four treatment arms as APT, CBT, GET and SSMC rather than APT plus SSMC, CBT plus SSMC, GET plus SSMC and SSMC alone*.

#### Secondary objectives

The secondary analyses are exploratory but we will be guided by previously published findings.

(1) Do different treatments have differential effects on outcomes (i.e. fatigue versus physical disability)?

(2) What baseline factors (other than randomised treatment) predict a reduction in (i) fatigue, (ii) disability in all participants?

(3) Are there differential predictors of response to APT, CBT, GET, and SSMC (i.e. treatment-covariate interactions)?

(4) Are there changes in factors (time-dependent covariates) during the earlier stages of treatment that (after controlling for baseline overall and differential predictors) are associated with outcome 1 year after randomisation?

(5) Are the differences across treatment groups in the primary outcomes associated with similar differences in secondary outcomes (e.g. in global change, mood, quality of life and objective measures of physical activity)?

### Hypotheses of efficacy

(1) APT is more effective than SSMC alone in reducing (i) fatigue, (ii) reducing physical disability and in reducing (iii) both.

(2) CBT is more effective than APT in reducing (i) fatigue, (ii) disability and in reducing (iii) both

(3) GET is more effective than APT in reducing (i) fatigue, (ii) disability and in reducing (iii) both

(4) The *active *rehabilitation therapies (of either CBT or GET) are more effective than the *adaptive *approach of APT in reducing fatigue, physical disability and both

(5) CBT is more effective than SSMC in reducing (i) fatigue, (ii) disability and in reducing (iii) both

(6) GET is more effective than SSMC in reducing (i) fatigue, (ii) disability and in reducing (iii) both

Other secondary hypotheses will be stated pre-hoc in an Analysis Strategy document.

### Type of design

**Figure 1:**
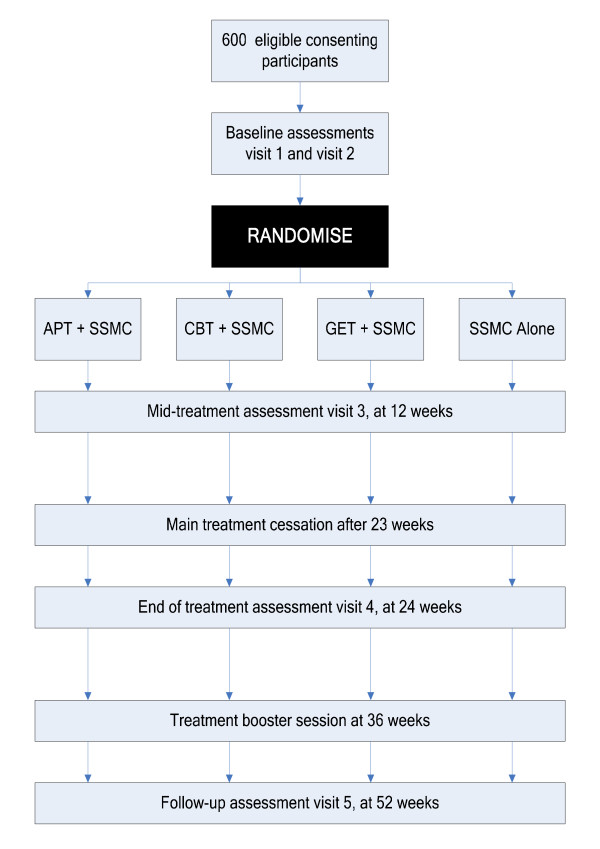
Table 1: Flowchart of trial design.

A four arm, randomised multi-centre parallel group controlled trial of patients who meet operationalised criteria for CFS/ME, with follow-up for 52 weeks (see Figure 1).

### Trial treatments – interventions and control

There are four treatment arms. SSMC is given to all participants. Three quarters will also receive one of the following supplementary therapies: APT, CBT or GET.

### Duration

Patients will be assessed for eligibility and those who are eligible and give consent will be randomly allocated to one of four treatments. Treatment will start as soon as possible after randomisation. The final outcome assessment will be at 52 weeks post randomisation.

### Number and source of participants

We will study 600 participants, recruited from new patient attenders, over approximately three years in six centres. All participants will be attending secondary care chronic fatigue clinics.

All centres have reported that they currently see a minimum of 100 new patients per year. We estimate that 60 will meet eligibility criteria, and we estimate that two thirds of these will agree to enter the trial, giving potentially a minimum of 40 participants per centre. In previous trials of both CBT and GET, only 7 and 15% of eligible participants refused to participate in GET trials [23,24] and 3, 10 and 26% of those eligible refused to participate in the three previous CBT trials [18,25,26]. We are therefore confident that recruitment is feasible and that the trial will recruit 600 participants over three years.

### Projected recruitment

Recruitment estimates are based upon 80% efficiency for the first three months rising to 100% efficiency by six months.

### Inclusion criteria

1. Both participant and clinician agree that randomisation is acceptable.

2. The participant has given written informed consent.

3. The participant meets operationalised Oxford research diagnostic criteria for CFS [2].

4. The participant's Chalder Fatigue Questionnaire score is 6 or more [27].

5. The participant's SF-36 physical function sub-scale score [28] is 65 or less.

6. The participant is aged at least 18 years old.

### Exclusion criteria

1. All potential participants will be screened for medical exclusions, by history and physical examination [1,2,4,29]. Appropriate investigations [4,29] will be undertaken by either the referring doctor or the centre doctors (checked by the research nurse) in the six months before baseline screening. Patients with a relevant alternative medical diagnosis will be excluded [2]. Investigations will be those recommended by the Royal Colleges' Report on CFS/ME and the CMO's working group report [4,29].

2. The research nurse (RN) will use a standardised psychiatric interview (the Structured Clinical Interview for DSM-IV – SCID) [30], under supervision by a participating centre PI or nominated deputy, to exclude those who are at significant risk of self-harm and those with psychiatric exclusions listed in the Oxford diagnostic criteria for CFS [2].

3. Patients who are considered by the RN, in discussion with their centre leader, to be unable to do one or more of the trial therapies or to complete all trial measures or for whom participation in the PACE trial would be inappropriate to their clinical needs (e.g. someone with significant post-traumatic stress disorder or borderline personality disorder).

4. Patients who have previously attended a PACE centre specialist fatigue clinic and received a course of treatment, from a specialist, considered to be similar to SSMC or any of the supplementary therapies of APT, CBT, or GET as delivered in the trial will be excluded from taking part in the trial.

### Screening/Baseline Procedures

Written informed consent will be taken before any trial related procedure takes place. Therefore PACE will utilise a two-stage consent/enrolment process. In the first stage the patient will consent to take part in the eligibility and baseline assessments and in the second stage the patient will consent to the full trial including randomisation, treatment and follow-up assessments. This has the added advantage of allowing one week's consideration by potential participants before consenting to the full trial.

### Data recording and Case Report Forms

Data will be recorded on Case Report Forms (CRFs). These will be completed by the patient for the self-report measures, and all other data will be collected and completed by the RN. The CRFs  will be checked for completeness and legibility by the RN before being entered onto the trial database by a local data manager (DM). Once data has been entered onto the local database, the data will be transferred to the senior data manager on the trial who will compare the hard copy CRFs with the database to check accuracy. S/he will check all the primary outcome variables and a randomly chosen 20 percent of the other variables. All CRFs for the first ten patients randomised per centre will be double checked. If there are any errors on primary outcomes, or greater than 1% errors of other variables, 100% data checks will be completed until the error rate ceases or drops. The database will not include the assigned treatments – these will be recorded in a separate database, in order for the statistician analysing the data to remain blind to treatment allocation.

### Initial screening for eligibility – visit 0 (clinic doctor)

New referrals to the outpatient clinics may be received from GPs or any other appropriate medical practitioner. Each clinic doctor will ensure that all consecutive new outpatients with a clinical diagnosis of CFS/ME are considered for the trial (i.e. if thought to be eligible they are told about the trial). Each centre leader will keep a trial log-book of every new chronic fatigue outpatient referral. This log book will detail each patient seen, whether or not they were referred for the trial and the reasons if not.

Where the patient is thought to be suitable by the clinic doctor (with a CFQ score of 6 or above and an SF-36 score of 65 or below), and the patient agrees to be assessed for eligibility, the clinic doctor will forward the patient's contact details to the RN. The clinic doctor will give the patient the trial Participant Information Sheet. The RN will contact the patient to arrange the first research visit (visit 1).

### Telephone assessment

The RN will contact patients within 24 hours of receipt of referral, who have been referred by the clinic doctor for the PACE trial, by telephone. The RN will check that the patient has received a Participant Information Sheet from the clinic doctor, and if they express interest in the trial, will arrange a date for the patient to attend the first research assessment interview (visit 1) as soon as possible (within one week of referral, but not more quickly than 48 hours after receipt of the Participant Information Sheet).

### Eligibility assessment and consent for assessment – visit 1

All of the following eligibility criteria must be fulfilled for the patient to participate:

1. The patient has a clinical diagnosis of CFS [2].

2. The patient does not have treatment needs that would make participation in the PACE trial inappropriate.

3. The patient is aged 18 years or above.

4. The patient can speak and read English at a level adequate for participation in the trial, as assessed by the RN. The reasons for this include the need to self-rate written primary and secondary outcomes using scales that have not been validated in non-English languages; the need to receive therapy that can be checked for quality and manual adherence; and the prohibitive cost of providing therapy in more than one language.

5. The Chalder Fatigue Questionnaire score is 6 or more [27].

6. The SF-36 physical function sub-scale score is 65 or less [28].

7. The Structured Clinical Interview for DSM-IV (SCID i/P; non-patient edition with psychotic screen) [30], will be used to exclude patients with psychiatric exclusions [2]. If a participant or patient is found to have a current psychiatric diagnosis on the SCID, the RN will inform the clinic doctor. All SCIDs will be audio-recorded for the purposes of quality control and RN supervision; the supervision being provided by the centre leader or their nominated deputy.

8. Patients who are considered by the RN in discussion with their centre leader to be unable to do one or more of the trial therapies or to complete all trial measures (travel expenses will be offered for therapy and research assessments).

9. There is no contra-indication to any of the treatments that might be provided in the trial.

10. Permission has been obtained to review medical notes.

In addition, the following assessments will be completed at baseline visit 1:

1. Participant demographic details will be collected (including date of birth, age, sex, ethnicity, marital or partner status, years of education, occupation)

2. Duration of CFS/ME (months)

3. Medical History

4. Co-morbid and current medical conditions

5. Current and specific membership of a self-help group (specific question)

6. Body Mass Index (BMI) (measure weight in kg and height in metres)

7. The six-minute walking test [31]

At the end of this visit the RN will give the participant the further baseline self-report questionnaires to complete at home and return at visit 2. These questionnaires are as follows:

1. The Chronic Disease Self-Efficacy measure [32]

2. The Work and Social Adjustment Scale [33]

3. Symptom Interpretation Questionnaire [34]

4. Physical Symptoms (Patient Health Questionnaire; PHQ-15) [35]

5. Exercise and Activity scale [36]

6. Jenkins Sleep Scale of subjective sleep problems [37]

7. The Hospital Anxiety and Depression Scale (HADS) [38]

8. The EuroQOL (EQ-5D) [39]

9. The RN will also fit the actometer [18] to the patient with an appropriate explanation and ask them to wear it until return on visit 2 or for one week (whichever is soonest). After visit 1 the research nurse will discuss the patient's potential eligibility with the centre leader.

### Eligibility assessment and consent for trial – visit 2

At visit 2 to the RN (after one week) the patient will return the actigraphy watch. If the patient meets all of the eligibility criteria and none of the exclusion criteria, understands the purpose of the trial and is willing to give informed consent to be randomised, treated and followed up, they will then sign the second consent form to participate in the full trial.

### Completion of baseline assessment

The following baseline assessments will be completed at visit 2:

1. Current medications and therapies (including complementary and alternative treatments)

2. The CDC criteria for CFS [1]

3. The London criteria for myalgic encephalomyelitis [40]

4. Presence or absence of fibromyalgia (using chronic widespread pain criteria only and not tender points) [41]

5. Preferred treatment group (single question)

6. The Client Service Receipt Inventory (CSRI), adapted for use in CFS/ME [42]

7. The self-paced step test of fitness [43]

8. The Borg Scale of perceived physical exertion, scored once immediately after the step test [44]

9. The actigraphy watch will be removed and the actigraphy data [18] (as initiated at visit 1 with the research nurse) will be downloaded.

### Randomisation and Enrolment procedure

Participants will be allocated to one of the four trial arms (ratio 1:1:1:1) by the Mental Health & Neuroscience Clinical Trials Unit (MH&N CTU) based at the Institute of Psychiatry. Allocation will be stratified by *centre*, *CDC Criteria *(met or unmet), *London Criteria *(met or unmet) and *depressive disorder *(major, minor depressive episode and dysthymia being present or absent) using minimisation with a random component [45]. The stratification on these criteria is to ensure equal proportions in each treatment arm. The first N cases (N will not be disclosed) will be allocated using simple randomisation to further enhance allocation concealment.

Once an eligible participant has completed the baseline assessment and given written informed consent, the RN will contact the MH&N CTU for treatment allocation by facsimile, giving the criteria needed for randomisation. Minimisation is carried out with a random component using a customised Microsoft Access database which will be used to hold the basic details collected to facilitate subsequent verification and to generate the allocation. Allocation is concealed because an independent group are responsible for this allocation. The confirmation of stratification details and treatment allocation will be communicated by email or facsimile to the RN within 24 hours. The RN sends back an acknowledgement of receipt to the CTU. This whole procedure is kept independent and separate from the trial statisticians. The RN will on the same day inform the participant of his/her treatment group in person or by phone, and will also inform the SSMC doctor and appropriate therapist. The therapist will contact the participant to arrange the first treatment appointment as soon as possible (within 5 working days). The SSMC doctor will also arrange to see the participant within one month of treatment allocation. The individual assignments will be available to the local team on a need-to-know basis, with the exception of the trial statisticians.

### Participant Identification Number

The participant identification number (PIN) will be a five digit number whereby the first two digits denote the centre and the remaining three denote the participant number by centre allocated in order of the patient entering the screening phase. Therefore every patient who consents to baseline and eligibility assessment will have a PIN, but not all will be randomised due to some being ineligible or not giving further consent.

### Randomised treatments

Apart from those receiving SSMC alone, all participants will be offered equal therapist time; 90 minutes in the first session, and 14 subsequent sessions of 50 minutes. The 15^th ^session will be a "booster" session given at week 36, thirteen weeks after the 14^th ^session, itself given at 23 weeks, which will be the last week for therapy. Therapy sessions 2 to 15 need not last the full 50 minutes if not required. If both therapist and participant believe that the next planned session is redundant because therapy is going so well, the next session may be omitted, with a note made as to the reasons why.

If the participant is unable to attend an appointment in person (e.g. due to feeling too disabled or due to intercurrent ill-health), and this cannot be re-arranged within five working days, and if agreed by both the therapist and participant, this session may be held over the telephone either at the pre-arranged time or within five working days of the original appointment. If the session does not take place within this time, the visit will be recorded as a DNA (Did Not Attend). Ideally, no more than four sessions of the first 14 sessions should be held in this way, and they should not be sequential. However, we believe it would be better that the participant receives some therapy rather than none at all and this will be judged on a per-participant basis. For this reason, if the choice is between not holding a session and a telephone session, a telephone session will always be offered even if there already have been four telephone sessions. This policy is supported by the results of one RCT and an open trial having suggested that two of the therapies (CBT and GET) delivered by telephone sessions following a face to face initial assessment is efficacious [46,47]. The fifteenth session will be held face-to-face, if at all possible, but even this may be held by telephone if the alternative is non-attendance.

We have chosen 15 sessions for all supplementary treatments on the basis of the previous trials of CBT and GET [18,23-26], as well as extensive clinical experience. RCTs of the least effective CBT and GET trials used 6 and 8 sessions [25,48]. Although one study of a pragmatic rehabilitation found that only 4 sessions were helpful [47], we suspect that this result may have been related to the lack of a 'treatment as usual' control group, and that more than four sessions are necessary to achieve change. A two year follow-up of this trial showed that the maximal face-to-face intervention had better efficacy by this time [19]. All interventions will be based on manuals, revised following feedback from both patients and therapists after piloting the manuals and therapies on patients outside of the trial.

### Adaptive Pacing Therapy

*APT *will be based on the illness model of CFS/ME as a currently undetermined organic disease, with the assumption that APT can improve quality of life, although not affect the core disease, other than providing the best conditions for natural recovery. APT is essentially an energy management approach, which involves assessment of the link between activity and subsequent symptoms and disability, establishing a stable baseline of activity using a daily diary, with advice to plan and pace activity in order to avoid exacerbations. Strategies include developing awareness of early warning of exacerbations; limiting demands; regular planned rest and relaxation, and alternating of different sorts of activities. The aim is to achieve optimal adaptation to the illness [4,16,17]. The patient charity *Action for M.E. *have helped in the design of the APT manual and have endorsed this version of pacing, which is based on what is published and what patients and clinicians have reported as helpful. Both therapists and participants will receive separate manuals.

### Cognitive Behaviour Therapy

*CBT *will be based on the illness model of fear avoidance, used in the three positive trials of CBT [18,25,26]. There are three essential elements: (a) Assessment of illness beliefs and coping strategies, (b) structuring of daily rest, sleep and activity, to establish a stable baseline of general activities, with a graduated return to normal activity, (c) collaborative challenging of unhelpful beliefs about symptoms and activity. Both therapists and participants will receive separate manuals.

### Graded Exercise Therapy

*GET *will be based on the illness model of deconditioning and exercise intolerance, used in the previous trials [23,24,47]. Therapy involves an assessment of physical capacity, establishing a stable baseline level of physical activity, negotiation of an individually designed home exercise programme with set target heart rates and times, and participant feedback with mutual planning of the next fortnight's exercise programme. Both therapists and participants will receive separate manuals.

### Standardised Specialist Medical Care

*SSMC *will be given to all participants. This will include visits to the clinic doctor with general, but not specific advice, regarding activity and rest management, such as advice to avoid the extremes of exercise and rest, as well as pharmacotherapy for specific symptoms and comorbid conditions. SSMC is standardised in the SSMC Doctor's Manual. As well as this, SSMC participants, like all other participants, will already have received the Patient Clinic Leaflet (PCL). The PCL is a generic leaflet explaining what CFS/ME is, its likely causes, and available treatments. There will be no additional therapist involvement. In particular there will be no diary monitoring with consequent advice. The number of SSMC outpatient sessions will be recorded, along with any treatments given for each participant by the SSMC doctor. Participants will be seen by their SSMC doctor a minimum of three times after randomisation, with the first SSMC appointment taking place as soon as possible after randomisation, and within one month. Further sessions will be determined by clinical need. Trial therapists, participants and general practitioners can request an unplanned clinical review by the SSMC doctor.

### Departures from randomised treatment

We will use the following strategies to minimise missing data in primary outcomes. Participants who drop out of treatment will be assessed as soon as possible, rather than waiting for the normal follow-up. Those who cannot attend clinic will be offered home assessments by the RN (or failing this assessment by telephone or by post), or centre leader as appropriate. If that is not achieved, we will seek to obtain outcome data by use of either postal or e-mail questionnaires, supplemented by telephone calls if necessary.

### DNAs from treatment

The therapist (if they have one) or SSMC doctor will contact the participant by telephone in the first instance to ascertain the problem of attendance, and will discuss the appropriate solution with the participant. Choices include a telephone session or a re-arranged face-to-face session, so long as the latter is within five working days. Alternatively the session stays a DNA and is recorded as such. If the participant considers that they are deteriorating the policy for this problem will be enacted.

### Clinician/Researcher withdrawal of participant from treatment

The reason for this will be recorded. When this occurs, the centre leader or nominee should assess the participant clinically within a week, and arrange appropriate care. Every effort will be made to obtain the two primary outcomes and the CGI (to assess illness progression), which should be scored in order to provide some outcome data. Such participants' data will be included in the trial analysis. If the participant will still consent to research (RN) follow-up, this will continue as normal.

### Participant withdrawal of consent to randomised treatment

In the first instance, the therapist (if they have one) or SSMC doctor will contact the participant by telephone to ascertain the reason for drop-out, if the participant is willing to share this, and will discuss the appropriate solution with the participant and then the centre leader. If the participant considers that they are deteriorating, but does not wish to talk to the therapist or SSMC doctor, the centre leader or nominee should contact them themselves.

If possible, the reason for withdrawal (e.g. adverse events, intercurrent illness, illness progression, inability to adhere, inability to attend regularly for treatment or assessment) should be ascertained. This information will be passed on to the other relevant members of the team and the trial manager (TM). The centre leader will ensure that every effort is made to obtain the primary outcome measures and the Clinical Global Impression (CGI) change score [49] from participants who drop out of treatment as soon as this occurs, even if they are not dropping out of the trial follow-up itself.

The centre leader or nominee will also ascertain whether consent is withdrawn from further trial treatment only or from both trial treatment and follow-up and in the latter case, whether the participant has given permission to retain data collected before treatment withdrawal for use at final analysis.

### Participant withdrawal of consent to research follow-up

If a participant withdraws consent for research (RN) follow-up during the trial, the centre leader or nominee should be informed on the same day, if possible. The centre leader or nominee will then contact the participant to find out why the participant wishes to withdraw from research follow-up, if they are willing to give a reason. The centre leader or nominee will also determine whether the participant has given permission to retain data collected before withdrawal for use at final analysis, or whether this information should be destroyed. No data from the latter participant will be used in analysis.

### Loss to follow-up

Permission will be sought from the Office of National Statistics (ONS) in England and the Information and Statistics Division (ISD) in Scotland, to track all participants randomised using NHS numbers. If a participant is lost to follow-up, the participant's GP will be contacted in the first instance, and if the participant has moved from the area, ONS (or ISD) will be contacted for details of the participant's new GP. This will only occur if the participant has given explicit consent (as detailed on the consent form) to allow this.

In all these situations the centre leader should inform the general practitioner and any referring doctor that their patient has withdrawn from either the trial or the trial treatment.

### Measures of treatment compliance/adherence

The SSMC doctor will record how many clinic outpatient sessions were attended, and how many were not attended during the 52 weeks by reviewing the medical notes.

If the participant has been receiving supplementary therapy, the therapist will record how many sessions/part sessions out of 15 were attended; whether they were face-to-face or telephone consultations and the durations of each session attended. At the end of therapy, the therapist will also score how well the participant adhered to the general therapy approach.

### Modification of trial treatment

Trial treatments will only be modified with the advice of the TSC, having been advised by the DMEC that a particular treatment arm is causing a consistent pattern of deterioration, or if there is another obvious and significant clinical necessity. The MREC will also need to approve any change in treatment.

### Additional therapy after the trial

Participants who are judged to require further therapy after their involvement in the trial has been completed, will be offered additional therapy. The choice of additional therapy will be agreed by the participant, clinic doctor and relevant therapist, and will start after the final follow-up interview (52 weeks after randomisation into the trial).

### Absence of a therapist

There will be occasions throughout the course of the trial when a therapist is absent due to annual leave, sickness, maternity leave or resignation. In these instances treatment delivery will be modified in order that a participant's therapy and the trial may continue uninterrupted. Three contingency plans have been devised to allow for a flexible approach to tackling this situation when it arises.

#### Therapy from a nearby centre

Local centre cover is delivered by a PACE therapist of the same discipline working in a nearby PACE centre.

#### Distant combined therapy

Distant therapy is delivered by a PACE therapist of the same discipline, whereby the therapist will conduct some visits face-to-face and the remainder by telephone. The participant at the same time may also be treated by a local cross-cover PACE therapist.

#### Local cross-cover therapy

Cross-cover therapy is delivered by a PACE therapist of a different discipline, whereby the cross-cover therapist learns a second PACE therapy to a competent level. They are supervised both by a distant centre PACE therapist of the appropriate discipline and a local therapist of the same discipline providing emergency assistance and assessment in case the patient has an intercurrent problem (e.g. pulls a muscle during GET).

#### Recruitment of a new therapist

In the case of resignation or maternity leave, the collaborating centre will seek to recruit a replacement therapist as quickly as possible.

It is recognised that there is a shortage of therapists working in the NHS and for this reason, the recruitment of staff of alternative appropriately qualified disciplines may also be considered. For example, an exercise physiologist may be recruited in place of a physiotherapist to deliver GET. There have been two randomised controlled trials of GET for CFS/ME provided by exercise physiologists, with positive outcomes [23,50]. In these instances the therapist will operate as a 'physiotherapy assistant' to a supervising physiotherapist. Similar alternative disciplines and supervision arrangements may also be considered for APT and CBT.

#### Changes to consent process

If a participant is to receive treatment from a therapist of either a different centre or a different discipline, the participant will give additional informed consent once it is clear that they understand this and are willing to receive their treatment in this way.

### Assessments and Procedures

#### Assessments

All participants will usually be assessed at the hospital. Those participants who cannot attend clinic will be offered home assessments (or failing this assessment by telephone or by post). Before the second and consequent RN assessments, self-rated measures will be posted to the participant prior to the visit and checked for completion at assessment by the RN. If a participant becomes too tired or ill to continue with the assessment, they will be offered the opportunity to complete the assessment on another day, within the next seven days.

Because we do not think it practically possible for the RN to remain blind to treatment group allocation, we will not attempt to achieve this. All our primary and secondary outcomes are therefore either self-rated or objective in order to minimise observer bias. Participants who drop out of treatment will be assessed for outcomes as soon as possible, rather than waiting for the normal follow-up.

When the participant does not attend a research interview, the RN should send the self-rated questionnaires to the participant's home address, with a stamped addressed envelope. If questionnaires are not received back within a week, the RN should arrange to visit the participant at home and oversee completion of the questionnaires. If necessary, only the primary outcomes and the CGI [49] (to assess deterioration) should be the minimum completed.

#### Long term follow-up

Permission will be sought from the participant to be contacted annually for follow-up information regarding the participant's health and employment status. The participant will also be invited to remain in contact so that the results may be disseminated to them once published.

### Measures

#### Primary outcome measures – Primary efficacy measures

Since we are interested in changes in both symptoms and disability we have chosen to designate both the symptoms of fatigue and physical function as primary outcomes. This is because it is possible that a specific treatment may relieve symptoms without reducing disability, or vice versa. Both these measures will be self-rated.

The 11 item Chalder Fatigue Questionnaire measures the severity of symptomatic fatigue [27], and has been the most frequently used measure of fatigue in most previous trials of these interventions. We will use the 0,0,1,1 item scores to allow a possible score of between 0 and 11. A positive outcome will be a 50% reduction in fatigue score, *or *a score of 3 or less, this threshold having been previously shown to indicate normal fatigue [27].

The SF-36 physical function sub-scale [29] measures physical function, and has often been used as a primary outcome measure in trials of CBT and GET. We will count a score of 75 (out of a maximum of 100) or more, *or *a 50% increase from baseline in SF-36 sub-scale score as a positive outcome. A score of 70 is about one standard deviation below the mean score (about 85, depending on the study) for the UK adult population [51,52].

Those participants who improve in both primary outcome measures will be regarded as overall improvers.

#### Secondary outcome measures – Secondary efficacy measures

1. The Chalder Fatigue Questionnaire Likert scoring (0,1,2,3) will be used to compare responses to treatment [27].

2. The self-rated Clinical Global Impression (CGI) change score (range 1 – 7) provides a self-rated global measure of change, and has been used in previous trials [45]. As in previous trials, we will consider scores of 1 or 2 as a positive outcome ("very much better" and "much better") and the rest as non-improvement [23].

3. The CGI change scale will also be rated by the treating therapist at the end of session number 14, and by the SSMC doctor at the 52-week review.

4. "Recovery" will be defined by meeting all four of the following criteria: (i) a Chalder Fatigue Questionnaire score of 3 or less [27], (ii) SF-36 physical Function score of 85 or above [47,48], (iii) a CGI score of 1 [45], and (iv) the participant no longer meets Oxford criteria for CFS [2], CDC criteria for CFS [1] or the London criteria for ME [40].

5. The Hospital Anxiety and Depression Scale scores in both anxiety and depression sub-scales [38].

6. The Work and Social Adjustment scale provides a more comprehensive measure of participation in occupational and domestic activities [33].

7. The EuroQOL (EQ-5D) provides a global measure of the quality of life [39].

8. The six-minute walking test will give an objective outcome measure of physical capacity [31].

9. The self-paced step test of fitness [43].

10. The Borg Scale of perceived physical exertion [44], to measure effort with exercise and completed immediately after the step test.

11. The Client Service Receipt Inventory (CSRI), adapted for use in CFS/ME [31], will measure hours of employment/study, wages and benefits received, allowing another more objective measure of function.

12. An operationalised Likert scale of the nine CDC symptoms of CFS [1].

13. The Physical Symptoms (Physical Health Questionnaire 15 items(PHQ15)) [35].

14. A measurement of participant satisfaction with the trial will also be taken at 52 weeks [53].

#### Adverse outcomes

Adverse outcomes (score of 5–7 of the self-rated CGI) will be monitored by examining the CGI at all follow-up assessment interviews [49]. An adverse outcome will be considered to have occurred if the physical function score of the SF-36 [28] has dropped by 20 points from the previous measurement. This deterioration score has been chosen since it represents approximately one standard deviation from the mean baseline scores (between 18 and 27) from previous trials using this measure [23,25]. Furthermore, the RN will enquire regarding specific adverse events at all follow-up assessment interviews.

#### Predictors

1. Sex

2. Age

3. Duration of CFS/ME (months)

4. 1 week of actigraphy [18] (as initiated at visit 1 with the research nurse)

5. Body mass index (measure weight in kg and height in metres)

6. The CDC criteria for CFS [1]

7. The London criteria for myalgic encephalomyelitis [40]

8. Presence or absence of "fibromyalgia" [41]

9. Jenkins sleep scale of subjective sleep problems [37]

10. Symptom interpretation questionnaire [34]

11. Preferred treatment group

12. Self-efficacy for managing chronic disease scale [32]

13. Somatisation (from 15 item physical symptoms PHQ sub-scale) [35]

14. Depressive disorder (major and minor depressive disorder, dysthymia by DSMIV) (from SCID) [30]

15. The Hospital Anxiety and Depression Scale (HADS) [38] combined score

16. Receipt of ill-health benefits or pension

17. In dispute/negotiation of benefits or pension

18. Current and specific membership of a self-help group (specific question)

#### Process variables

1. Step test of fitness [43]

2. Borg Scale of perceived physical exertion [44]

3. The symptom interpretation questionnaire [34]

4. Exercise and activity scale

5. PHQ symptom sub-scale

6. HADS scale combined score

#### Therapeutic input

1. At each RN assessment participants will be asked what other treatments they have been receiving (e.g. complementary and alternative therapies, prescribed and over-the-counter medicines).

2. The strength of the therapeutic alliance will be measured by the therapy integrity rating scale by an independent and blinded observer [53].

3. The differentiation of the supplementary therapies will be measured blind to treatment group by an independent observer [53].

#### Plausibility of therapy

After the first treatment session, all participants will be asked to fill in a brief measure of how plausible their treatment appears to them.

#### Economic costs

The CSRI [42] will retrospectively record service utilisation for the six months prior to the baseline assessment, for the period between baseline and 24 weeks, and then for the period from 24 weeks to 52 weeks. A comprehensive range of services will be included so that in addition to being able to determine the resource implications to the NHS, we will also have information on the impact that treatment has on other parts of the care system as well as on informal carers. The ability to engage in employment, education and work in the home are frequently affected by CFS/ME and the CSRI will collect data on these activities. Service use will be valued by attaching appropriate unit costs from national sources (e.g. Netten et al, 2003 [54]) as well as intervention costs specifically calculated for the study.

#### Adverse Events

Adverse events (AE) are any clinical change, disease or disorder experienced by the participant during their participation in the trial, whether or not considered related to the use of treatments being studied in the trial.

#### Serious Adverse Events (SAEs)

A Serious Adverse Event will be defined according to usual clinical trial definitions and will be reported to the appropriate authorities in the standard manner. If there is any doubt in the minds of the RN and the centre leader as to whether the AE is a serious AE, the centre leader will obtain a second opinion from one of the PIs.

#### Serious Adverse Reactions (SARs)

A Serious Adverse Reaction can be defined as: An SAE that is considered to be a reaction to one of the supplementary therapies or a drug prescribed as part of SSMC.

#### Reporting serious adverse events and reactions (SAEs and SARs)

In the event of an adverse event (AE), the centre leader or nominee will judge the seriousness of the event, the relationship to a trial supplementary therapy or SSMC prescribed treatment, clinical severity and the expectedness of the event. All SAEs must be reported by the RN to the SSMC doctor (or SSMC doctor to the RN), centre leader or nominee (e.g. another centre leader), and the trial manager immediately the RN or SSMC doctor learns of the SAE, regardless of the relationship to trial treatment. Reporting of SAEs and SARs will be carried out according to normal regulatory requirements.

#### Non-serious adverse events and reactions

Non-serious adverse events or reactions will be assessed by the RN at each follow-up assessment interview. A risk assessment has been undertaken, and we have concluded that the therapies are of low risk to participants. Non-serious adverse events will be reported according to the usual regulatory requirements.

#### Follow-up after adverse events

After an SAE or SAR, a decision will be made by the centre leader as to whether the participant should be withdrawn from either their randomised treatment or from the trial, or need an alteration in their SSMC. Arrangements will be made by the centre leader for further assessment and management as required. Advice from the participant's GP, other health professionals or relevant local authorities will be sought for any instance of an SAE or SAR where further external advice is required. The RN will provide the centre leader and TM with a one month follow-up report on all SAEs and SARs. Further monthly reports should be provided in the absence of resolution. These reports will be communicated to the DMEC and MREC via the TM or trial statistician, and by the RN to the local Research and Development (R&D) office.

#### Safety of participants

There is a discrepancy between patient organisation reports of the safety of CBT and GET and the published evidence of minimal risk from RCTs. Surveys by *Action for M.E. *of its members suggest that people becoming worse with these treatments is caused by either rigidly applied programmes that are not tailored to the patient's disability, or by improperly supervised programmes [13-15]. PACE treatment manuals minimize this risk by being based on mutually agreed and flexible programmes that vary according to the patient's response. The RN will also carefully monitor for any adverse effects of the treatments.

#### Policy for deteriorating participant or one who drops out of treatment

The following policy will be enacted by the centre leader for any participant who is considered, or considers themselves, to be deteriorating, or has dropped out of treatment. The centre leader or delegated professional will undertake a detailed clinical assessment, at home if necessary, following which they will be offered appropriate help.

#### Recruitment, randomisation and retention

The right of the patient to refuse to participate in the trial without giving reasons must be respected. Those recruiting and randomising participants will rigorously maintain a position of equipoise and employ explanations that are consistent with this [55]. All the participating clinicians regard all the four treatments as potentially effective and are of the view that most patients seen will accept randomisation if it is fully and openly explained. Some patients are initially sceptical about treatment effectiveness but are willing to accept any of these recommended treatments as long the treatment is appropriately explained and delivered. Therefore, we do not anticipate a difficulty either in acceptability of the proposed treatments, with recruitment into the trial, or acceptance of randomisation. We emphasise that we make this statement based on our having completed six trials of treatment for CFS/ME. After the patient has entered the trial, the clinic doctor must remain free to give alternative treatment to that specified in the protocol, at any stage, if he/she feels it to be in the best interest of the patient.

#### Compliance

The trial will be conducted in compliance with the Declaration of Helsinki, the trial protocol, MRC Good Clinical Practice (GCP) guidance, the Data Protection Act (1998), the Multi-centre Research Ethics Committee (MREC) and Local Research Ethics Committees (LREC) approvals and other regulatory requirements, as appropriate. The final trial publication will include all items recommended under CONSORT.

#### Sponsor

The main sponsor is Barts and the London, Queen Mary School of Medicine and Dentistry.

#### Name of person/s authorised to sign the final protocol and protocol amendments for the sponsor

• Chair of the Trial Steering Committee, Professor Janet Darbyshire.

• Professor Stephen Stansfeld (on behalf of the Sponsor).

• The three principal investigators.

#### Research Ethics Approval (MREC)

Ethical approval for the PACE trial was given by the West Midlands MREC (reference number MREC/02/7/89). Local REC approvals have been sought and obtained as required.

#### Indemnity

Each centre taking part in the trial will seek local approval and indemnity through their NHS R&D department. As an automatic consequence of this, local NHS indemnity will apply to the PACE trial. Details of local indemnity arrangements can be obtained through each centre's NHS R&D department.

### Analyses

#### Assumptions

The existing evidence does not allow precise estimates of improvement with the trial treatments. However the available data suggests that at one year follow up, 50 to 63% of participants with CFS/ME had a positive outcome, by intention to treat, in the three RCTs of rehabilitative CBT [18,25,26], with 69% improved after an educational rehabilitation that closely resembled CBT [43]. This compares to 18 and 63% improved in the two RCTs of GET [23,24], and 47% improvement in a clinical audit of GET [56]. Having usual rather than specialist medical care allowed 6% to 17% to improve by one year in two RCTs [18,25]. There are no previous RCTs of APT to guide us [11,12], but we estimate that APT will be at least as effective as the control treatments of relaxation and flexibility used in previous RCTs, with 26% to 27% improved on primary outcomes [23,26]. We propose that a clinically important difference would be between 2 and 3 times the improvement rate of SSMC.

#### Power analyses

Our planned intention to treat analyses will compare APT against SSMC alone, and both CBT and GET against APT. Assuming α = 5% and a power of 90%, we require a minimum of 135 participants in the SSMC alone and APT groups, 80 participants in the GET group and 40 in the CBT group [57]. However these last two numbers are insufficient to study predictors, process, or cost-effectiveness. We will not be able to get a precise estimate of the difference between CBT and GET, though our estimates will be useful in planning future trials. As an example, to detect a difference in response rates of 50% and 60%, with 90% power, would require 520 participants per group; numbers beyond a realistic two-arm trial. Therefore, we will study equal numbers of 135 participants in each of the four arms, which gives us greater than 90% power to study differences in efficacy between APT and both CBT and GET. We will adjust our numbers for dropouts, at the same time as designing the trial and its management to minimise dropouts. Dropout rates were 12 and 33% in the two studies of GET [23,24] and 3, 10, and 40% in the three studies of rehabilitative CBT [18,25,26]. On the basis of our own previous trials, we estimate a dropout rate of 10%. We therefore require approximately 150 participants in each treatment group, or 600 participants in all. Calculation of the sample size required to detect economic differences between treatment groups requires data of cost per change in outcome, which is not currently available.

#### Unblinding

All research and therapy staff and participants are unblinded to treatment allocation of individual participants. Therefore there will be no need for unblinding during the trial. The one exception is the trial statisticians who are blind to treatment allocation (coded A, B, C, D), as will be the DMEC, in order to take actions on the basis of the unblinded data alone.

#### Analysis plan

A full Analysis Strategy will be developed, independently of looking at the trial database, and before undertaking any analysis. This paper summarises the analysis plan.

#### Primary analyses of efficacy

The primary analysis will be pragmatic, based on intention to treat, and will utilise all available follow-up data from all randomised participants. The primary binary outcomes of response on the fatigue and physical function sub-scales (comparing proportions with categorical adverse deterioration with this scale as well) and both and a combined response with will be analysed by logistic regression adjusted for centre with contrasts for:

(1) APT *vs*. SSMC alone,

(2) APT *vs*. CBT,

(3) APT *vs*. GET,

(4) Trend across SSMC alone, APT, and CBT/GET combined,

(5) CBT *vs*. SSMC alone,

(6) GET *vs*. SSMC alone.

Participants not followed to one year will be classed as non-responders unless they show a consistent pattern of outcome across assessments at 10, 24, and 39 weeks or whenever the last assessment is obtained.

#### Secondary analyses of efficacy

The secondary continuous outcomes will be analysed by repeated measures analysis of variance using a linear mixed model with AR(1) covariance structure, and including centre, depressive disorder, CDC and London criteria and baseline values as covariates. The same contrasts as those specified for the primary outcomes will be extracted. A summary measure, the area under the curve, will also be reported.

A secondary, per protocol, analysis restricted to participants who complete a minimum of 12 weeks of treatment (representing the mid point in therapy time), will also be performed.

Further secondary sensitivity analyses will be used to assess the robustness of conclusions for missing primary outcomes; these will employ repeated binary outcomes, multiple imputation, and imputation analysing all possible outcomes [58].

Loss to follow-up, departures from randomised treatment protocols, and the prevalence of serious adverse events, will be reported at 13, 26, 39, and 52 weeks from randomisation.

Results from all analyses will be summarised as differences between percentages or means together with 95% confidence limits (CL). The significance level for all analyses of primary outcome variables will be P = 0.05 (two-sided); for secondary outcome variables, P = 0.01 (two-sided) unless profiles of response can be specified in advance.

Prior to writing the Analysis Strategy a consensus will be reached on the profiles of response for each secondary outcome within each of the four treatment groups.

#### Predictions and process of treatment

Associations between post-treatment outcomes and both predictor and process variables (including demographic, illness duration, and other putative clinical indicators) will be examined using multiple linear and logistic regression modelling techniques, including a limited examination of interactions both amongst pairs of predictors and between predictors and treatment groups. We anticipate that the sample size will be sufficient to identify important general predictors from a random-split, training set of two thirds (~400), with partial validation in the remainder, used as a test set. Shrinkage techniques (to allow for over-optimism in variable selection) will be applied in the development of a prognostic model to be applied to participants outside the trial.

#### Economic analyses

The main economic evaluation will be a cost-effectiveness analysis conducted from a societal perspective, examining comprehensive costs (treatment and service costs plus lost productivity) and the two primary efficacy measures (fatigue and physical function). Cost-effectiveness acceptability curves will be plotted as necessary. A supportive cost-consequences analysis will be conducted, examining comprehensive costs alongside all (primary and secondary) efficacy measures. To inform special interests, evaluations will also be conducted from the perspectives of the NHS, and also by using utility scores in the cost-effectiveness analysis (computed from either the EQ-5D [39] or the WSAS [33], there being arguments for and against each as the basis for health-related quality of life measurement).

#### Monitoring

The principal investigators, centre leaders and participants will permit trial-related monitoring, audits, ethics committee review and regulatory inspections by providing direct access to source data/documents.

#### Independent overseers

The Data Monitoring and Ethics Committee (DMEC) will advise on the frequency of reviews of the data on the basis of accrual and event rates.

The role of the independent Trial Steering Committee (TSC) is to provide overall supervision for the trial and safeguard its integrity. Executive authority for the continuation of the trial lies with the TSC.

#### Confidentiality

All data collected will be regarded as confidential and securely stored.

#### Quality assurance and quality control

Quality assurance and control will be ongoing throughout the trial.

#### Therapists' compliance with treatment manuals

Therapist compliance with treatment manuals will be monitored in two ways. 1) All therapists will receive a minimum of monthly telephone individual supervision sessions, and face-to-face group and individual supervision at least four times a year, depending on supervisory needs. All therapy sessions will be video/audio-recorded. Some recordings will be used by trainers/supervisors to provide feedback to therapists on competence and treatment fidelity, which will happen particularly in the first few months of a therapist starting to treat participants. Any significant deviations from the manual will be noted and feedback given to the therapist. Therapist competence will be measured by the relevant therapy leaders. Therapists will be allowed to treat trial participants once they have been approved as competent. 2) Two recorded sessions per therapist will be randomly chosen and assessed blindly and independently by an assessor to assess adherence to manual defined therapy.

#### SSMC doctors' adherence with SSMC manual

All SSMC doctors will receive training in use of the SSMC manual. All SSMC sessions will be audiorecorded. Some recordings will be used by centre leaders (using other centre leaders when the centre leader is providing SSMC) to provide feedback to doctors on competence and treatment fidelity, which will happen particularly in the first few months of a doctor starting to treat participants. Any significant deviations from the manual will be noted and feedback given to the doctor. Two recorded sessions per doctor will be randomly chosen and assessed blindly and independently by an assessor to assess adherence to manual defined treatment. In addition, this will be particularly done for any doctor who routinely sees participants more than five times in the twelve months of the study.

#### Participant non-adherence with treatment

Participant non-adherence with treatment will be measured both by recording attendance and by therapist ratings of adherence to therapy.

#### Database quality

The senior data manager will be responsible for checking the quality of the Trial Master Database (TMD), and will send local centre data managers query forms as necessary.

#### Data Monitoring and Ethics committee

Reports to DMEC and the main analysis itself (as far as possible) will be compiled blind to allocated treatment. DMEC reports will simply label treatments as A, B, C or D. DMEC may request unblinding only if they have serious concerns about any of the treatments. The unblinding would be handled by a third statistician independent of the TMG. The DMEC can recommend premature closure of the trial to the TSC. The circumstances for this need to be agreed by the DMEC and TSC, but we suggest the only likely scenario is if one of the trial treatments is shown to cause significant and consistent deterioration in a significant number of participants (to be quantified at the meeting of the DMEC). If one treatment arm does show consistent and reliable evidence of causing serious adverse reactions in participants, then consideration of closing that particular arm of the trial will be given. The DMEC will be asked to keep a close eye on any consistent pattern of deterioration of participants.

## Discussion

The PACE trial will be the largest randomised trial of available treatments for CFS/ME. It will provide important information about efficacy, adverse events, cost-effectiveness, process and predictors. This will inform patients, their carers, healthcare providers and commissioners which treatments are most useful for which patients, and provide information regarding the essential process of both recovery and improvement from CFS/ME.

### Current Study Status

The PACE trial opened to recruitment in March 2005.

## List of Abbreviations

**AE **Adverse Event

**AfME **Action for M.E.

**APT **Adaptive Pacing Therapy – in this protocol the abbreviation 'APT' refers to Adaptive Pacing Therapy given with Standardised Specialist Medical Care

**CBT **Cognitive Behaviour Therapy – in this protocol the abbreviation 'CBT' refers to Cognitive Behaviour Therapy given with Standardised Specialist Medical Care

**CFS **Chronic fatigue syndrome

**CFS/ME **Chronic fatigue syndrome/myalgic encephalomyelitis or encephalopathy – Official term for the illness as described in the 'Working group report to the Chief Medical officer' (2002) and the MRC RAG report (2003)

**CMO **Chief Medical Officer for England

**CRF **Case Report Form

**CSO **Chief Scientist's Office for Scotland

**CTU **Clinical Trials Unit

**DM **Data Manager

**DMEC **Data Monitoring and Ethics Committee

**DH **Department of Health

**DM **Data Manager

**DWP **Department for Work and Pensions

**ELCMHT **East London and City Mental Health Trust

**GCP **Good Clinical Practice

**GET **Graded Exercise Therapy – in this protocol the abbreviation 'GET' refers to Graded Exercise Therapy given with Standardised Specialist Medical Care

**ISD **Information and Statistics Division

**ISRCTN **International Standard Randomised Controlled Trial Number

**LREC **Local Research Ethics Committee

**ME **Myalgic encephalomyelitis/encephalopathy

**MRC **Medical Research Council

**MREC **Multi-centre Research Ethics Committee

**ONS **Office for National Statistics

**PACE P**acing, graded **A**ctivity and **C**ognitive behaviour therapy: a randomised **E**valuation

**PI **Principal Investigator

**PCL **Patient Clinic Leaflet

**PIN **Participant Identification Number

**PIS **Participant Information Sheet

**PTM **Participant Treatment Manual

**R&D **Research & Development – also referred to as NHS R&D

**RN **Research Nurse

**SAE **Serious Adverse Event

**SAR **Serious Adverse Reaction

**SL&M **South London & Maudsley NHS Trust

**Sponsor **Individual/organisation responsible for the initiation, management/financing of a clinical trial

**SSMC **Standardised Specialist Medical Care

**SUSAR **Suspected Unexpected Serious Adverse Reaction

**TCMF **Trial Centre Master File

**TM **Trial Manager

**TMD **Trial Master Database

**TMG **Trial Management Group

**TSC **Trial Steering Committee

## Competing interests

PDW has done voluntary and paid consultancy work for the Departments of Health and Work and Pensions and legal companies and a re-insurance company. MCS has done voluntary and paid consultancy work for government and for legal and insurance companies. TC has done consultancy work for insurance companies, is the author of Coping with Chronic Fatigue published by Sheldon Press and co-authors Overcoming Chronic Fatigue with Mary Burgess published by Constable and Robinson. RW and JD have no competing interests to declare.

## Authors' contributions

PDW, MCS and TC are the co-principal investigators, conceived the study and developed the design of this trial. RW is one of the trial statisticians and participated in the design of the study. JD is the trial manager and coordinator and contributed to trial design and protocol development. All co-authors read and approved the final article.

## Pre-publication history

The pre-publication history for this paper can be accessed here:


